# Vacuum distillation residue upgrading by an indigenous *bacillus cereus*

**DOI:** 10.1186/2052-336X-11-18

**Published:** 2013-07-16

**Authors:** Mitra Sadat Tabatabaee, Mahnaz Mazaheri Assadi

**Affiliations:** 1 Department of Biology, Faculty of Science, Islamic Azad University, Central Tehran branch, Tehran, Iran; 2 Environmental Biotechnology, Biotechnology Department, Iranian Research Organization for Science and Technology, Tehran, Iran

**Keywords:** Vacuum distillation residue, Contaminated soil, Crude oil, *Bacillus cereus*, Upgrading

## Abstract

**Background:**

Biological processing of heavy fractions of crude oils offers less severe process conditions and higher selectivity for refining. Biochemical Processes are expected to be low demand energy processes and certainly ecofriendly.

**Results:**

A strain of biosurfactant producing bacterium was isolated from an oil contaminated soil at Tehran refinery distillation unit. Based on selected phenotypic and genotypic characteristic including morphology, biochemical proprety, and 16 SrRNA sequencing identified as a novel strain of *Bacillus cereus* (JQ178332). This bacterium endures a wide range of pH, salinity and temperature. This specific strain utilizes both paraffin and anthracene as samples of aliphatic and polycyclic aromatic hydrocarbons. The ability of this bacterium to acquire all its energy and chemical requirements from Vacuum Distillation Residue (VR), as a net sample of problematic hydrocarbons in refineries, was studied. SARA test ASTM D4124-01 revealed 65.5% decrease in asphaltenic, 22.1% in aliphatics and 30.3% in Aromatics content of the VR in MSM medium. Further results with 0.9% saline showed 55% decrease in asphaltene content and 2.1% Aromatics respectively.

**Conclusion:**

Remarkable abilities of this microorganism propose its application in an ecofriendly technology to upgrade heavy crude oils.

## Background

Heavy crude oils (bitumen) are extremely viscous and make up of asphaltene, waxes, resins and polycyclic heteroaromatic hydrocarbons containing sulfur and nitrogen. These properties of heavy crude oil and also strict laws of environmental protection make it important for refiners to convert heavier oils into lighter and more valuable products efficiently in very strict operational conditions. Depleting light crude oil reservoirs, scientific interests increase to utilize the vast sources of unconventional crude oil which are heavy and full of problematic components [1-3].

Vacuum distillation residue (VR), the end product of crude oil distillation including high molecular weight PAH, asphaltenic components and waxes [4,5] is a net sample of problematic components of heavy crude oil for laboratory experiments [6,7].

Since biological processing of heavy crude oil may offer less severe processing conditions in refineries and higher selectivity to specific reactions to increase net distillates, it is proposed that the microorganisms capable to biodegrade heavy fractions of VR, could present an applicable opportunity for upgrading heavy crude oils [8-10].

Bactria able to biodegrade various components of petroleum hydrocarbons such as poly-nuclear aromatic hydrocarbons (PAHs), like anthracene, monoaromatic hydrocarbons such as toluene, or aliphatic hydrocarbons such as *n*-alkanes, are widely reported, particularly from petroleum-contaminated sites [3,11-14]. But there are few reports on isolates that can alter several problematic petroleum components simultaneously, which are all find in VR.

The microbial decontamination of petroleum-polluted soils seems to be an efficient, economic, and versatile alternative to physicochemical treatments. Several abiotic and biotic parameters including the conditions for microbial degradation activity (e.g., presence of nutrients, oxygen, pH, and temperature), the quality, quantity, and bioavailability of the contaminants (e.g., particle size distribution), and the soil characteristics, which are hardly to be controlled in the in situ condition, affect the rate of microbial degradation of hydrocarbons in soils [15,16].

Therefore the bacteria with high physicochemical endurance and degradation ability could be a proper choice not only in bioremediation but also in other aspects of oil industry, like heavy oil bio-upgrading or microbial enhanced oil recovery.

Bacteria have several means to overcome the difficulty of complex hydrocarbon uptake. For instance, as bioavailability is the main limitation factor for biodegradation of petroleum hydrocarbons [12,17] due to their chemical structure [18], bacteria produce surfactant to increase bioavailability of PAHs, desorption and solubility of them in the aqueous phase [18,19] and consequently enhance the oil mobility and improving the biodegradation rates [20-22]. Another cell modification leading to new ecotypes may include amendments of the cell envelope to tolerate solvents [23].

Thus to select a useful bacterium for industrial use and bioremediation propose, a sample of soil contaminated with oil for decades in distillation section of Tehran refinery has been screened for bacteria able to degrade and utilize VR ,the most complex structure of petroleum, as its sole source of carbon and energy. Such a bacterium with high environmental endurance and biodegrading a wide range of hydrocarbons could be useful in remediation, heavy crudes bio-upgrading and many other petroleum industry applications.

## Methods

A sample of soil contaminated with crude oil and VR was taken from the field in the distillation unit of Tehran refinery. The soil sample was continuously enriched for a month with VR of Ahvaz crude oil as the sole source of carbon and energy [24,25] in aminimal salt medium (MSM) consisting of K_2_HPO_4_,0.5(g); NH_4_Cl,1(g); Na_2_SO_4_,2(g); KNO_3_,2(g); CaCl_2_ .6H_2_O, 0.001(g);mgSO_4_ .7H_2_O,1(g); Feso_4_ . 7H_2_O, 0.001(g) in 1 liter distilled water pH7.0 [5]. It was kept under constant agitation at 150 rpm in 30°C, that was the ambient temperature at the sampling site, for a month with the objective of enriching those microorganisms which can utilize the heavy structure of VR as their sole source of carbon and energy [26]. The sample was enriched because indigenous bacteria in the soil can degrade a wide range of target constituents of the petroleum, but their population and efficiency are affected when any toxic contaminant is present at high concentrations [27,28]. This adapted soil indigenous bacterial consortium with VR consisted of several culturable bacteria, isolated by standard plate count method.

### Bacterial selection

Among the isolates the best grown bacteria on VR was chosen for further identification.

The growth and VR-degrading ability of the bacterium studied inminimal salt medium as described, supplementing with 5% VR as the sole source carbon and energy [29].

### Preculture prepration

Before each inoculation a primary preculture was prepared as following: 5 ml of the bacterial suspension with optical density equal 1(using T80 UV/VIS spectrometer) enriched in 100 ml nutrient broth; after 48 hours incubation at 30°C, that was inoculated in MSM supplemented with 5% VR and 0.1% glucose as a growth stimulator and agitated for 48hours in 150 rpm at 30°C, then this preculture was used as inoculum in different experiments. Initially it was inoculated in VR-MSM medium at 30°C, 150 rpm for 20 days [29,30].

To study the ability of the selected bacteria to degrade both aliphatic [16] and aromatic contents of VR, 1% Paraffin [29] and 50 mg/l anthracene [31] both from Merck company production, have been served separately in MSM broth as sole sources of carbon and energy for 48 hrs at 30°C in 150 rpm.

### Sulfur free culture

The sulfur source of the MSM medium was eliminated (BSM) to investigate the ability of bacteria to acquire its sulfur requirements from VR, 1 ml of bacterial suspension has been inoculated in 100 ml BSM and the growth rate was determined by spectrophotometer at 600nm during 20 days of agitation at 150 rpm ,at 30°C. BSM consists of 4(g) K_2_HPO_4_, 4(g) Na_2_HPO_4_, 2(g) NH_4_Cl, 0.2(g)mgCl.6H_2_O, 0.001(g) CaCl_2_.2H_2_O, 0.001(g) FeCl_3_.6H_2_O in 1 liter distilled water pH7.0 [32].

The probability of utilizing VR as the source of all chemical requirements for bacterial growth was studied by inoculation of 1ml bacterial suspension (0.5 McFarland) in Saline (physiologic serum), supplemented with 5%VR and shaking for 20 days at 30°C at 150 rpm. Saline was used to avoid bacterial destruction at the inoculation stage [29].

Bacterial growth in each media was studied by measuring the bacterial cell population (viable count) and optical density changes at 600 nm with spectrophotometer (T80 UV/VIS spectrometer). All the experiments were done in triplicate.

### Biosurfactant production

Biosurfactant production was proved by Blood hemolysis [33], drop collapse [19,34,35] and oil spreading techniques [36]. The emulsification ability of the isolate was measured using Krepsley method [17,37]. Emulsification activity was performed both with Iranian light crude oil and n-hexadecane. They were added to bacterial broth in a ratio of 3:2 and then shook for 1minute. After 24 hours rest the height of the emulsified compound was divided by the total height of the system multiplied by 100. Experiments were performed in triplicate for each compound [37].

### Hydrophobicity

The bacterial adhesion to hydrocarbons (BATH) assay was used to determine changes in cell surface hydrophobicity during growth and adaptation with VR in a twenty-day period. The BATH assay was performed as described in literature [21,38,39]; however, the washing steps and centrifugation were omitted to avoid cell surfaces damaging [40]. Briefly, 4 ml of bacterial suspension (106 to 107 cells ml - 1) was vortexed with 1 ml of ***n***-hexadecane for at least 2 min. Then the aqueous and hydrocarbon phases were separated in 15 min. One milliliter of the aqueous phase was carefully sampled with a Pasteur pipette. The concentration of cells left in the water phase was determined using Spectrophotometer. Hydrophobicity (expressed as a percentage) was calculated as follows:

$$\left[\left(a-b\right)/a\right]\times 100,$$

where *a* is the initial cell concentration in the aqueous phase and *b* is the cell concentration in the aqueous phase after partitioning [39,41]. This assay was once done in nutrient broth and simultaneously in the MSM supplemented with VR after incubation period at 30°C to determine the hydrophobicity alteration of the bacteria in contact with VR.

### Chemical analysis

VR was extracted with 10 ml Dichloromethane due to its volatility and ability to dissolve a wide range of organic compounds, from aquatic phase of the growth media in a separator y funnel in triplicate. After the solvent's evaporation at room temperature, samples were analyzed. The residue were fractionated into their four principal components - saturates, aromatics, resins, and asphaltenes - commonly known as SARA fractions [42]. The standard method ASTM D4124-01 (2002) was applied in this study [43]. The sample was first separated into *n*-heptane insoluble asphaltenes and *n*-heptane-soluble petrolenes. Then petrolenes are absorbed on calcined GC-20 alumina and fractioned further into saturates, aromatics and resins by downward solvent elution in a glass chromatographic column. Each assay was performed three times and the average is reported.

### Identification

The selected bacterium was characterized by standard morphological, Gram-reaction, physiological and biochemical techniques [44] and preliminarily identified as a *Bacillus sp.* The selected *Bacillus* was identified molecularly through 16SrRNA sequencing. fD1 and rD1 primers (Sinagene/Tehran/Iran) were used for PCR: fD1^*^:5’ccgaattcgtcgacaacagagtttgatcctggctcag3’, rD1^*^:5’cccgggatccaagcttaaggaggtgatccagcc3’.

Then the PCR product [45] has been cloned in *P Bleuescript Sk* plasmid. White colonies had been chosen and after confirmation of the presence of 16SrRNA sequence in that, the fragment has been sequenced using fD1 and rD1 primers.

### Physicochemical endurance

Effect of temperature on the isolate was studied using 100mlmineral salt medium with 5 ml VR and 1ml preculture at pH6.8 on shaker at 150 rpm for 48 hrs. Then the pH and salinity endurance of the bacteria in MSM broth at 150 rpm for 48 hrs was respectively studied [31].

All experiments were done in triplicate at the adaptation temperature (30°C) and in a short period of 20days (for biodegradation). To analyze the biodegradation an un-inoculated flasks with VR were served as control under each experiment condition.

## Results

Bacterial growth inminimal medium supplemented with vacuum residue was evident from the significant increase in the population as compared to control by measuring cell density at 600 nm and alternate colony counting, both in 20 days of incubation period [46]. The bacterium which could more efficiently transform the rough appearance of VR in shorter time and could tolerate the toxicity of it with better growth rate in different growth media, was chosen for further identification.

**Figure 1:**
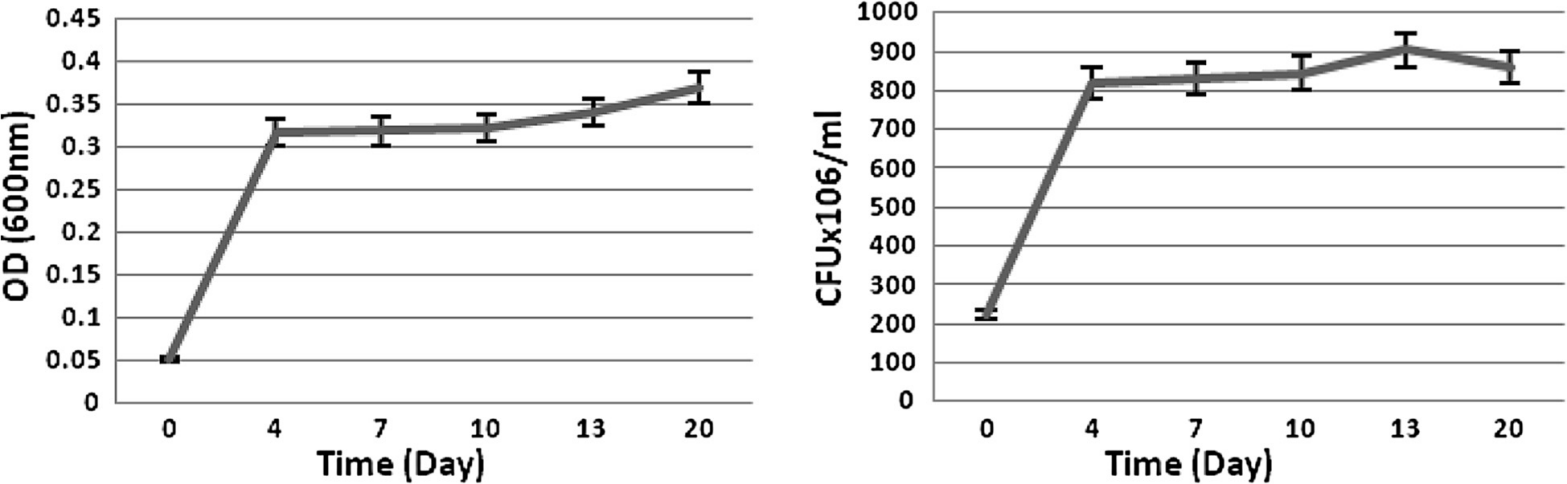
Bacterial growth in MSM broth.

The selected isolate grew continuously and the turbidity increased during 20 days (Figure 1), and the rough and rigid appearance of VR changed during the incubation period into small globular structures. Values represent the average of three experiments.

**Figure 2:**
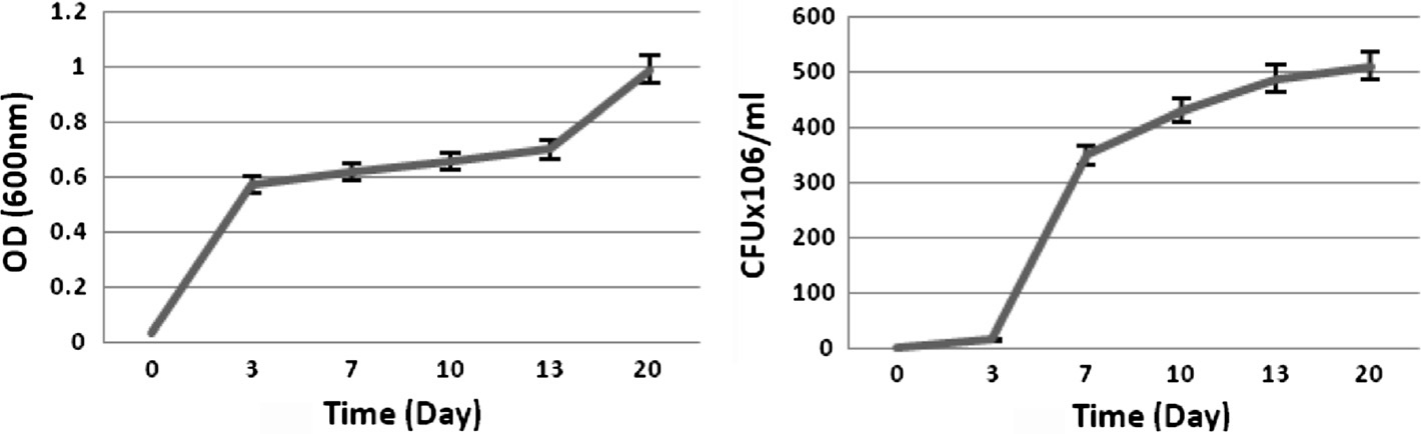
Bacterial growth in BSM broth.

**Figure 3:**
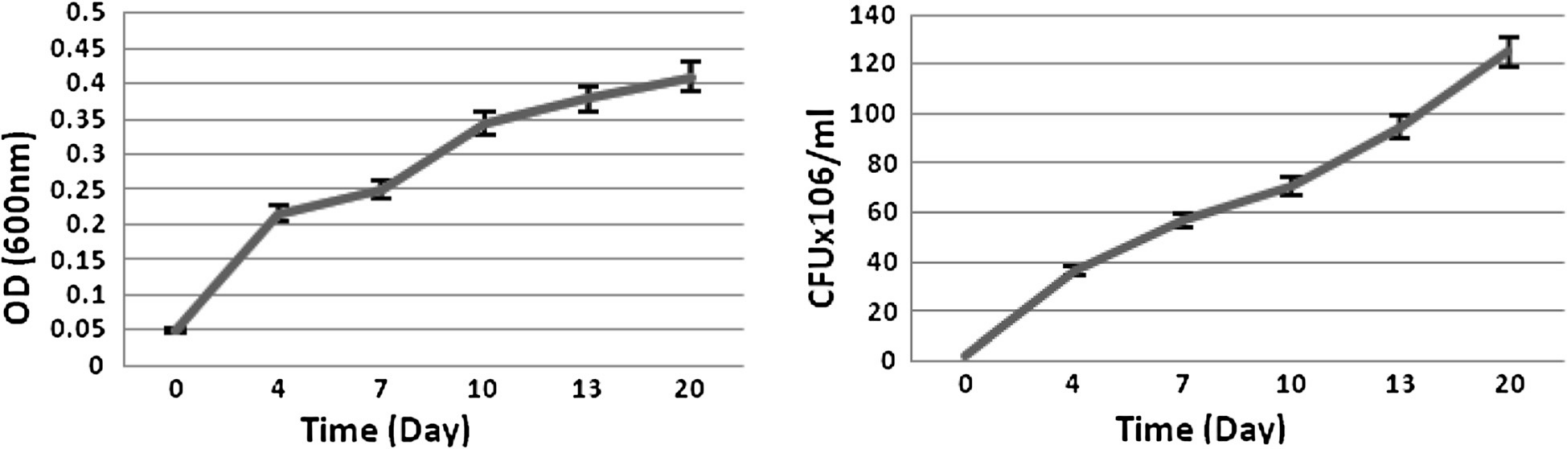
Bacterial growth in saline.

Bacterial growth was studied in sulfur free medium (Figure 2). A considerable turbidity was reported in saline supplemented with VR after shaking at 150rpm and 30°C (Figure 3).

**Figure 4:**
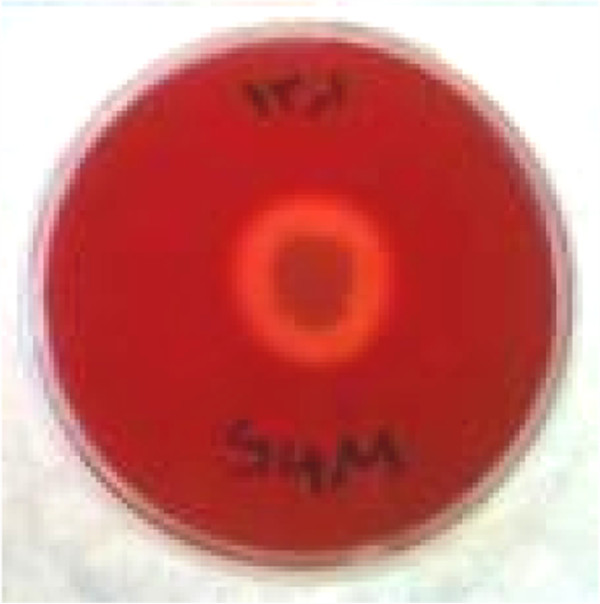
Bacterial hemolysis plate.

The emulsification activity of the bacterium was 10.2% in n-hexadecane and 7% in crude oil. The drop collapse test, oil spreading technique with 50 mm diameter clear zone and RBC hemolysis with over 75 mm diameter clear zone in 24 hours confirmed the biosurfactant production [19,47] (Figure 4).

The hydrophobicity of the bacterial cells was 10.99% before incubation with VR and it had increased to 16.67%. The percentages are the average of triplicate experiments.

**Figure 5:**
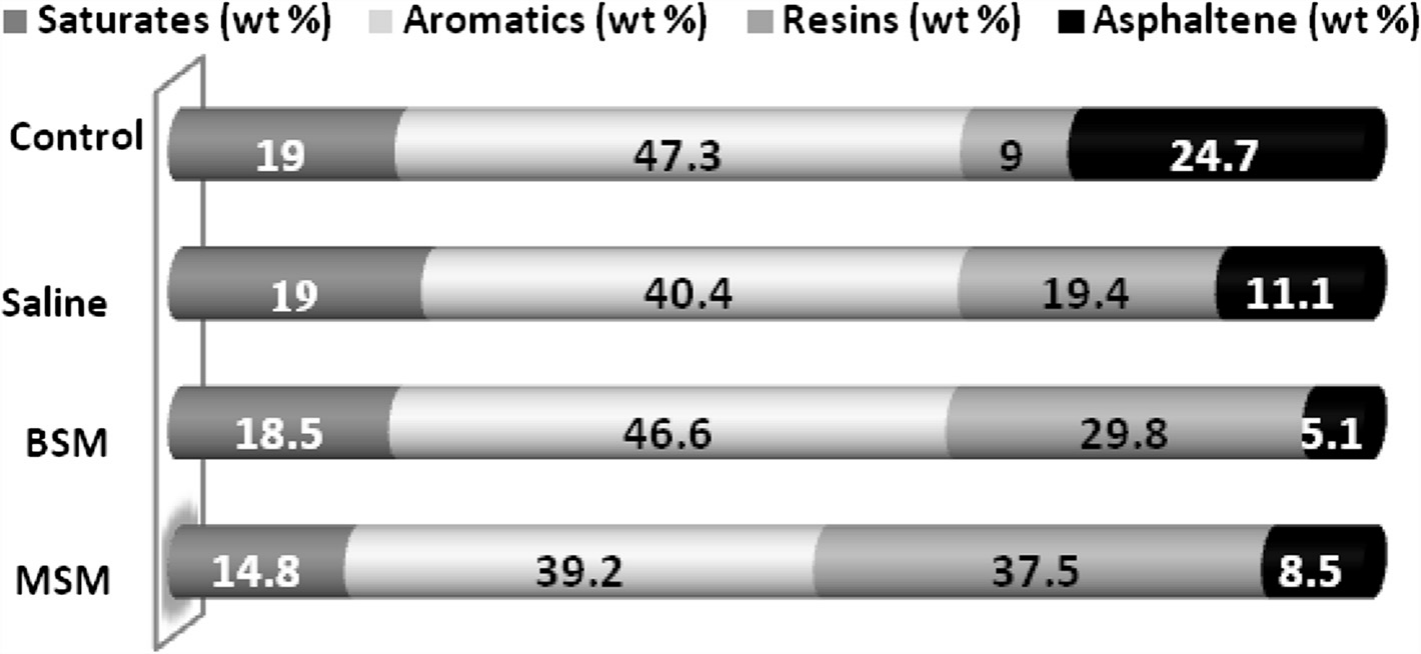
SARA content weight percent.

The result of VR chemical analysis for the percentage of alkanes, aromatics, asphaltenes and resins, using SARA test after 20 days treatment with the bacterium in different media, is illustrated in Figure 5.

The selected bacterium was able to grow in a wide range of pH from 5.5 to 8, salinity up to 3% and temperature from 20°C to 55°C.

Bacterial growth was observed both in MSM broth supplied with anthracene or with paraffin by medium turbidity after 48 hours.

Biochemical (Table 1) and Molecular analysis defined the selected bacterium as a novel strain of *Bacillus cereus.* This bacterium accession number in GenBank is appeared as JQ178332.

**Table 1:** Biochemical tests of selected bacterium

**Tests**	**Selected bacterium**
Gram staining	+
Spore staining (malachite green)	+
Catalase	+
Citrate	_
SIM	S (-) , Movement (+)
Starch hydrolysis	+
lecithinase	+

## Discussion

This study indicated that there are bacteria able to biodegrade complexes of both aliphatic and aromatic hydrocarbon in extreme nutritional conditions along with each other in only presence of water. This finding could be a useful approach in environmental biotechnology.

Several studies have already proved the ability of Bacilli in degradation of various structures of hydrocarbons [30,46,48,49] but there are rare information of vacuum distillation residue, containing both asphaltene and wax, degradation.

Molecular analysis of the selected, spore forming, biosurfactant producing bacterium as a novel strain of *Bacillus cereus* (JQ178332) in this study, ascribed this bacterium with 94% similarity to *bacillus cereus Q1*(CP000227.1), previously described as a thermopile bacterium by Xiong et.al 2009 from deep oil reservoir [49].

As VR content analysis illustrates, there is 22.1% decrease in saturate alkanes, 30.3% in Aromatics, and 65.5% in Asphaltenes percentage and remain of resin as the last bacterial invaded component when VR treated with selected bacterium in 30°C in 150 rpm shaking for 3 weeks in MSM [14].

## Conclusion

The high physicochemical endurance of *Bacillus cereus* isolated from petroleum contaminated soil with the ability to utilize both aliphatic and complex aromatic structures of distillation residual substances as its sole source of carbon and energy, and the particular finding of this research that revealed the remarkable ability of the bacterium to use VR as the only source of all required chemicals for growth along with surfactant production, make this bacterium a unique option for industrial use, particularly in bioremediation, bio-upgrading and biorefining processes. This bacterium with ability to biodegrade and utilize heavy fractions of vacuum distillation residue as its sole source of carbon and energy can be useful in petroleum biological processing with less severe condition to increase net distillates.

## Competing interests

The authors declare that they have no competing interests.

## Authors’ contributions

MMA was the project principle investigation and supervisor to MST during her PhD thesis. MST was the PhD scholars in above said project. The work is a part of her PhD thesis. Both authors read and approved the final manuscript.

## Authors’ information

The first author contributed as PhD scholar and second author as supervisor, to this work.
